# Optimized nucleus isolation protocol from frozen mouse tissues for single nucleus RNA sequencing application

**DOI:** 10.3389/fcell.2023.1243863

**Published:** 2023-09-28

**Authors:** Marie-Albane Minati, Angeline Fages, Nicolas Dauguet, Jingjing Zhu, Patrick Jacquemin

**Affiliations:** ^1^ de Duve Institute, Université Catholique de Louvain, Brussels, Belgium; ^2^ Flow Cytometry and Cell Sorting Facility (CYTF), de Duve Institute, Université Catholique de Louvain, Brussels, Belgium; ^3^ Ludwig Institute for Cancer Research, Université Catholique de Louvain, Brussels, Belgium

**Keywords:** nucleus isolation, frozen tissues, placenta, pancreas, snRNASeq

## Abstract

The single cell RNA sequencing technique has been particularly used during the last years, allowing major discoveries. However, the widespread application of this analysis has showed limitations. Indeed, the direct study of fresh tissues is not always feasible, notably in the case of genetically engineered mouse embryo or sensitive tissues whose integrity is affected by classical digestion methods. To overcome these limitations, single nucleus RNA sequencing offers the possibility to work with frozen samples. Thus, single nucleus RNA sequencing can be performed after genotyping-based selection on samples stocked in tissue bank and is applicable to retrospective studies. Therefore, this technique opens the field to a wide range of applications requiring adapted protocols for nucleus isolation according to the tissue considered. Here we developed a protocol of nucleus isolation from frozen murine placenta and pancreas. These two complex tissues were submitted to a combination of enzymatic and manual dissociation before undergoing different steps of washing and centrifugation. The entire protocol was performed with products usually present in a research lab. Before starting the sequencing process, nuclei were sorted by flow cytometry. The results obtained validate the efficiency of this protocol which is easy to set up and does not require the use of commercial kits. This specificity makes it adaptable to different organs and species. The association of this protocol with single nucleus RNA sequencing allows the study of complex samples that resist classical lysis methods due to the presence of fibrotic or fatty tissue, such as fibrotic kidney, tumors, embryonic tissues or fatty pancreas.

## 1 Introduction

To better understand the complexity of organs, many researchers have focused their studies on the characterization of the transcriptome of the cells that make up tissues of the studied organs. Nowadays, this characterization frequently relies on a technique called single cell RNA sequencing (scRNA-seq). This method allows to investigate the transcriptomic profile of a unique cell, after tissue dissociation into a single-cell suspension, and thus to classify and characterize cell types related to the studied organ. This technique has considerably increased the knowledge of organ biology. However, scRNA-seq has some limitations regarding organs or tissues difficult to dissociate into single-cell suspension (Habib et al., 2017) or incompatible with single-cell approach (Van Hauwaert et al., 2021). Moreover, scRNA-seq requires the use of fresh tissue. Single-nucleus RNA sequencing (snRNA-seq) can overcome these problems and represent an alternative to scRNA-seq. This technique analyzes the nuclei rather than the whole cells. Focusing the analysis on nuclei avoids the need to obtain a single-cell suspension and therefore allows to perform the analysis on frozen tissues or organs difficult to dissociate. In addition, snRNA-seq protects against potential changes in the transcriptomic profile resulting from enzymatic cell dissociation method (Grindberg et al., 2013; Wu et al., 2019).

The placenta, the first organ to be developed, is a key organ for the developmental process in mammals. This organ represents the interface between the maternal tissue and the embryo creating a link between the mother and the embryo which ensures nutrient transport and gas exchange (Knipp et al., 1999). Although it is known that unhealthy placenta leads to developmental problems of fetuses and so, to congenital disease or mortality, this organ is understudied (Winterhager et al., 2018). Very few transcriptomic data are available on healthy/unhealthy placenta. Yet these data are important for our understanding of cell and tissue function of this organ. Transcriptomic analysis on human or mouse placenta were initially performed with microarray analysis or RNA sequencing analysis on bulk placenta. Although interesting, these experiments have provided only a limited knowledge of the different placental cell populations. Single-cell RNA sequencing could help to better understand this issue; however, placenta displays a highly complex tissue architecture (although some differences exist between mammal species), making it difficult to obtain a single cell suspension with a good viability. To better understand human placental diseases, researchers use genetically engineered mouse (GEM) models. The use of GEM brings a new limitation of scRNA-seq, since all embryos of the litter do not have (in most cases) the desired mutation, and genotyping takes time. To overcome these limitations, the use of snRNA-seq is a solution, as it allows to perform the experiment on frozen tissues giving the opportunity to analyze the genotype of embryos and to select the desired embryos, before sending samples for sequencing.

Other organs are also likely to be problematic when performing scRNA-seq experiments. This is the case of the pancreas, which, in connection with its exocrine role involved in digestive function, contains a very high quantity of RNases. Indeed, during the dissociation of the organ to obtain the suspension of single cells, these RNases can contribute to strongly degrade the pancreatic RNAs. Furthermore, pathologies of the pancreas are associated with significant infiltration of adipocytes into the pancreatic parenchyma (called lipomatosis). This is the case of the pancreatic fat accumulation and fat replacement in diabetes, nonalcoholic fatty pancreas disease and Shwachman–Bodian–Diamond syndrome (Levin et al., 2015; Catanzaro et al., 2016; Zeng et al., 2022). The abnormal presence of adipocytes inside pancreatic parenchyma having metabolic repercussions (Filippatos et al., 2022; Wagner et al., 2022), and murine pancreas being suitable for the study of human pancreatic pathologies (Dolenšek et al., 2015; Lorberbaum et al., 2020), several research teams developed murine models representative of pancreatic diseases inducing lipomatosis (Augereau et al., 2016; Quilichini et al., 2019). These have contributed to a better understanding of the underlying mechanisms. At this stage, a more detailed understanding could still be provided by scRNA-seq studies of these models. However, due to specific properties of adipocytes, notably a high lipid content and a fragile plasma membrane, scRNAseq analyzes were found to be inadequate, here also favoring a snRNAseq approach (Sun et al., 2020; Gupta et al., 2022; Michelotti et al., 2022).

Faced with the difficulties of performing scRNA-seq experiments on these two organs, we used a snRNAseq approach to characterize our mouse models. This approach led us to develop a protocol for isolating nuclei from these two organs. This general protocol, which can also be used on other organs, is presented below.

## 2 Material and equipment

### 2.1 Animals

All procedures described below were performed with the approval of the animal welfare committee of the UCLouvain. Mice received humane care according to the criteria listed by the National Academy of Sciences. Mice used in this study were maintained in an enriched CD1 background. About the embryo study, male and female mice were mated together to obtain vaginal plug (the morning of vaginal plug detection is considered as embryonic day E0.5). Pregnant female was sacrificed 13 days after the vaginal plug detection (E13.5). Concerning the study of pancreas, 7-month-old mice were used.

### 2.2 Reagents and enzymes

- Bovine serum albumin (BSA) (Sigma, Belgium; Cat. no.: A3311-50G)
- DAPI (Life Technologies Europe, Belgium; Cat. no.: D1306)
- Dulbecco’s phosphate-buffered saline (DPBS) (Lonza, Belgium; Cat. no.: BE17-512F)
- NP-40 (Merck, Belgium; Cat. no.: 11332473001)
- Nuclease-Free water (Promega, Netherlands; Cat. no.: MC1191)
- RNaseOut (Life technology Europe, Belgium; Cat. no.: 10777019)
- Trypan blue (Merck, Belgium; Cat. no.: T8154)

### 2.3 Equipment

• 0.22 μm-filters (Fisher Scientific, Belgium; Cat. no.: 43075)
• 40 μm-cell strainers (Fisher Scientific, Belgium; Cat. no.: 431750)
• 1.5 mL-Sterile Eppendorf tubes (Eppendorf, Belgium)
• 50 mL-Polypropylene Falcon tubes (Fisher Scientific, Belgium)
• 250 mL-Pyrex bottles (Fisher Scientific, Belgium)
• 96-well PCR plates (Bio-Rad, Belgium; Cat. no.: HSP9655)
• Automated cell counter (Bio-Rad, Belgium; Cat. no.: TC20)
• Cell sorter machine (BD Biosciences, Belgium; Cat. no.: FACS ARIAIII)
• Fluorescence microscope (Zeiss, Belgium; Cat. no.: Axiovert 200)
• Spinning disk confocal microscope (Zeiss, Belgium)
• Magnetic bar and agitator
• Nuclease-free tips and pipettes (Westburg, Netherlands)
• Optical microscope (Zeiss, Belgium, Axiovert 40C)
• DNA BioAnalyzer system (Agilent Genomics, Belgium, Agilent 2100)
• Sterile dissection tools (i.e., scissors, forceps, and clamps) (Fine Science Tools, Germany)
• Sterile Petri dishes (Fisher Scientific, Belgium)

### 2.4 Reagent set-up

• Dissolve the required amount of solutes in distilled water to prepare stock solutions listed in Supplementary Table S1.
• Pass each solution through a 0.22 mm filter. Store the solutions at 4°C.
• The day before the experiment, prepare the lysis buffer and wash buffers, and store buffers at 4°C.
• The day of the experiment, keep the two buffers on ice.

### 2.5 Equipment set-up

• Before proceeding to dissection, prepare a tank with liquid nitrogen.
• Put Petri dishes on ice with cold PBS inside.
• Clean the benchtops and pipettes with 70% ethanol and RNase decontamination solution.
• Clean the homogenizer with 70% ethanol, RNase decontamination solution following by RNase-free water. Let it dry.
• Pre-cool the centrifuge to 4°C.
• Label 1.5 mL Eppendorf, and 50 mL tubes (number of tubes = number of conditions).

## 3 Stepwise procedure

**FIGURE 1:**
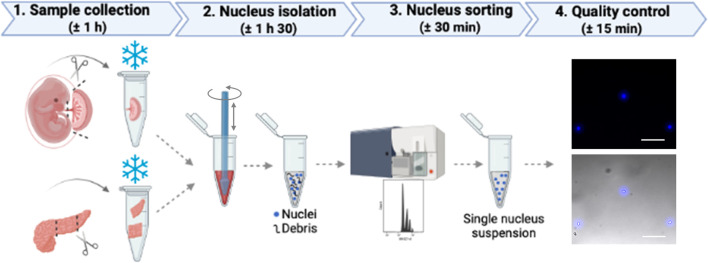
**Schematic workflow.** To obtain a single nucleus suspension from mouse placenta or pancreas, there are four major steps that include tissue collection followed by nucleus isolation, nucleus sorting, and a final quality control step to assess the shape of nuclei.

Figure 1 shows the general scheme of placenta and pancreas collection.

### 3.1 Placenta collection—±1 h

**FIGURE 2:**
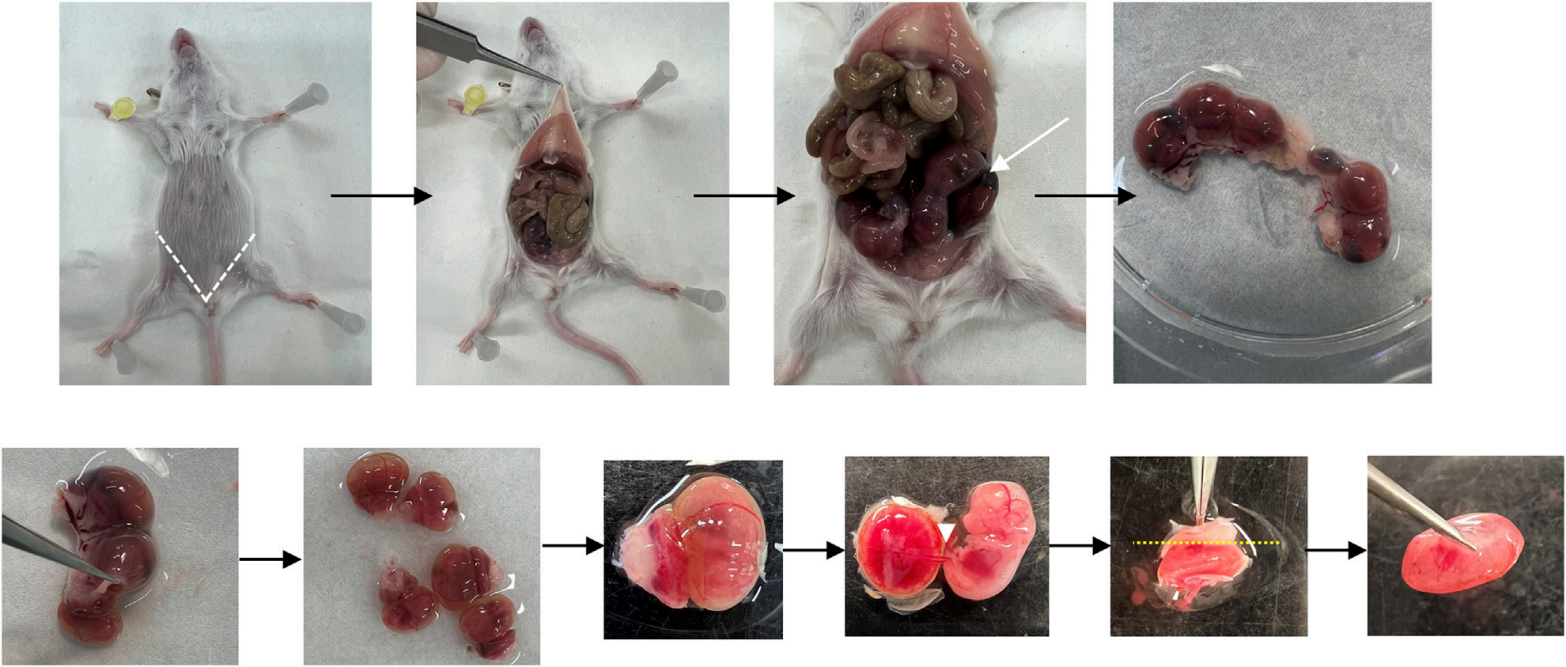
**Placenta collection**. Representative pictures showing the placenta collection. The white dotted lines locate the incisions made to obtain the placenta samples for the study. The white arrow shows uterus with embryos. The white arrowhead shows the umbilical cord. The yellow dotted lines locate the incisions made to remove the outermost layer of the decidua.

Figure 2 shows a scheme of the main steps of placenta collection.

1. Euthanize the pregnant mouse by cervical dislocation.
2. Place the mouse on a dissection tray and sterilize the abdomen with 70% ethanol.
3. Open the abdomen by two incisions (in V) at the bottom of abdomen (near the genital parts) and open the abdominal cavity using forceps to have access to the embryos.
4. Collect the uterus containing embryos and put it in a Petri dish containing cold PBS.
5. Remove all embryos from uterus by cutting the uterine tissue with scissors and put embryos in a new Petri dish containing cold PBS.
6. Cut the junction between placenta and extra-embryonic membranes and remove the yolk sac and allantois using forceps.
7. Cut the umbilical cord to separate the placenta from the embryo.
  OPTIONAL STEP: Cut the embryo tail and put it in a 1.5 mL Eppendorf to perform later the genotyping of each embryo.
8. Remove the outermost layers of the decidua by cutting the white part of the placenta.

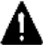
 CRITICAL STEP: Be careful to not disrupt the cells of the labyrinth or junctional zone.
9. Put the placenta in 1.5 mL tube and put it rapidly in liquid nitrogen.

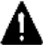
 CRITICAL STEP: Placenta need to be frozen very rapidly to avoid degradation of the tissue.

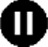
 PAUSE STEP: After snap froze, the placenta can be stored at −80°C for few months.
  OPTIONAL STEP: Perform the genotyping of embryos.

### 3.2 Pancreas collection—± 10 min

**FIGURE 3:**
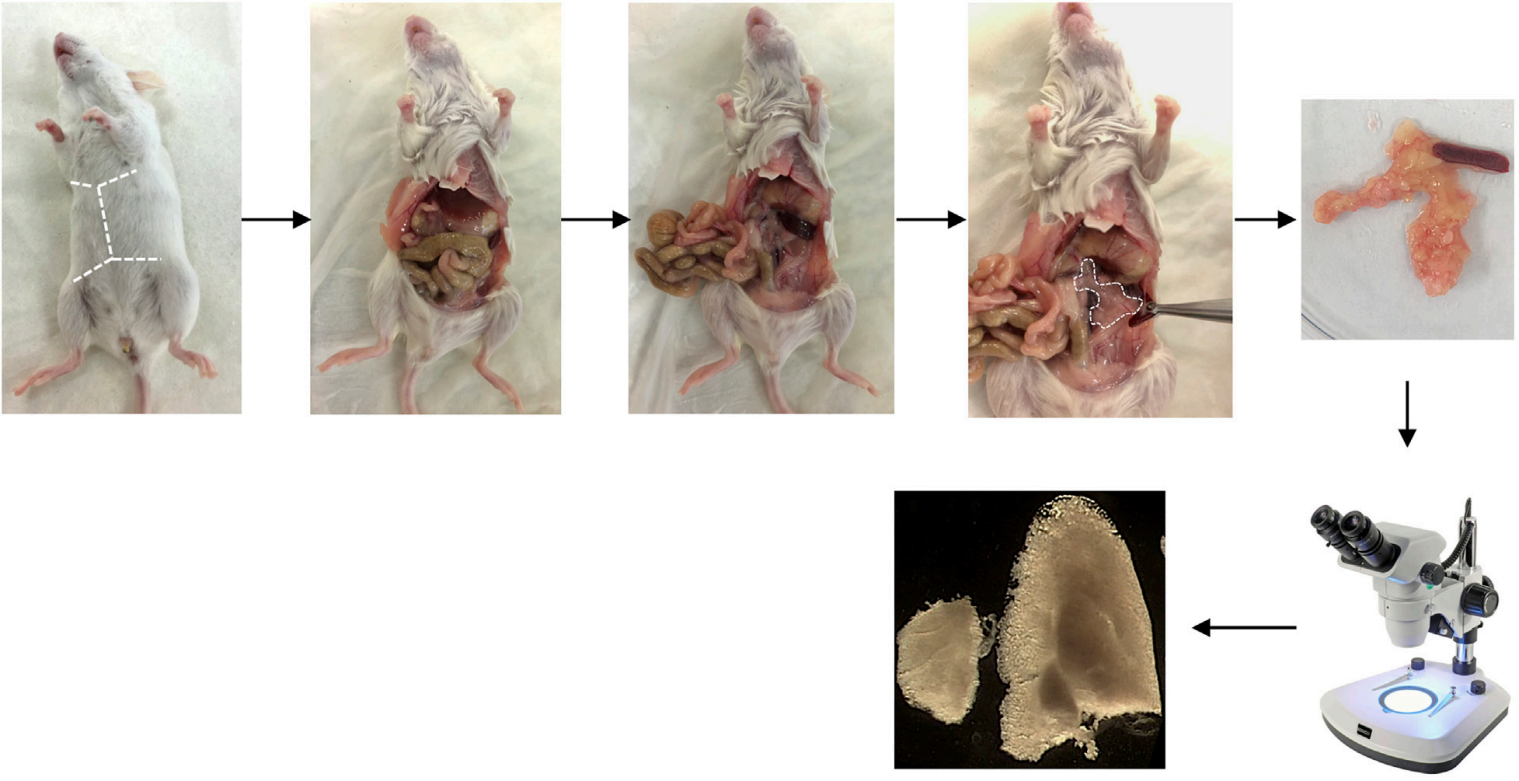
**Pancreas collection**. Representative pictures showing the pancreas collection. The dotted lines locate the incisions initially performed to dissect the pancreas.

Figure 3 shows the main steps of pancreas collection.

1. Euthanize the mouse by cervical dislocation.
2. Place the mouse on a dissection tray and sterilize the abdomen with 70% ethanol.
3. Open the abdomen by two incisions (in V) at the bottom of abdomen (near the genital parts) and open the abdominal cavity using forceps.
4. Shift intestines on the left of the body to give a better access to stomach, pancreas, and spleen.
5. Collect the pancreas by gently dissociating it from the adhesions with duodenum, stomach and spleen and put it in a Petri dish containing cold PBS.
6. Place the Petri dish under a stereomicroscope to start microdissection of pancreatic lobes.

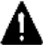
 CRITICAL STEP: Pancreas containing high RNAse activity, microdissection must be performed quickly to prevent RNA degradation.
7. Put the samples in 1.5 mL tube and put it rapidly in liquid nitrogen.

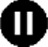
 PAUSE STEP: After snap froze, pancreatic samples can be stored at −80°C for few months.

### 3.3 Nucleus isolation

**FIGURE 4:**
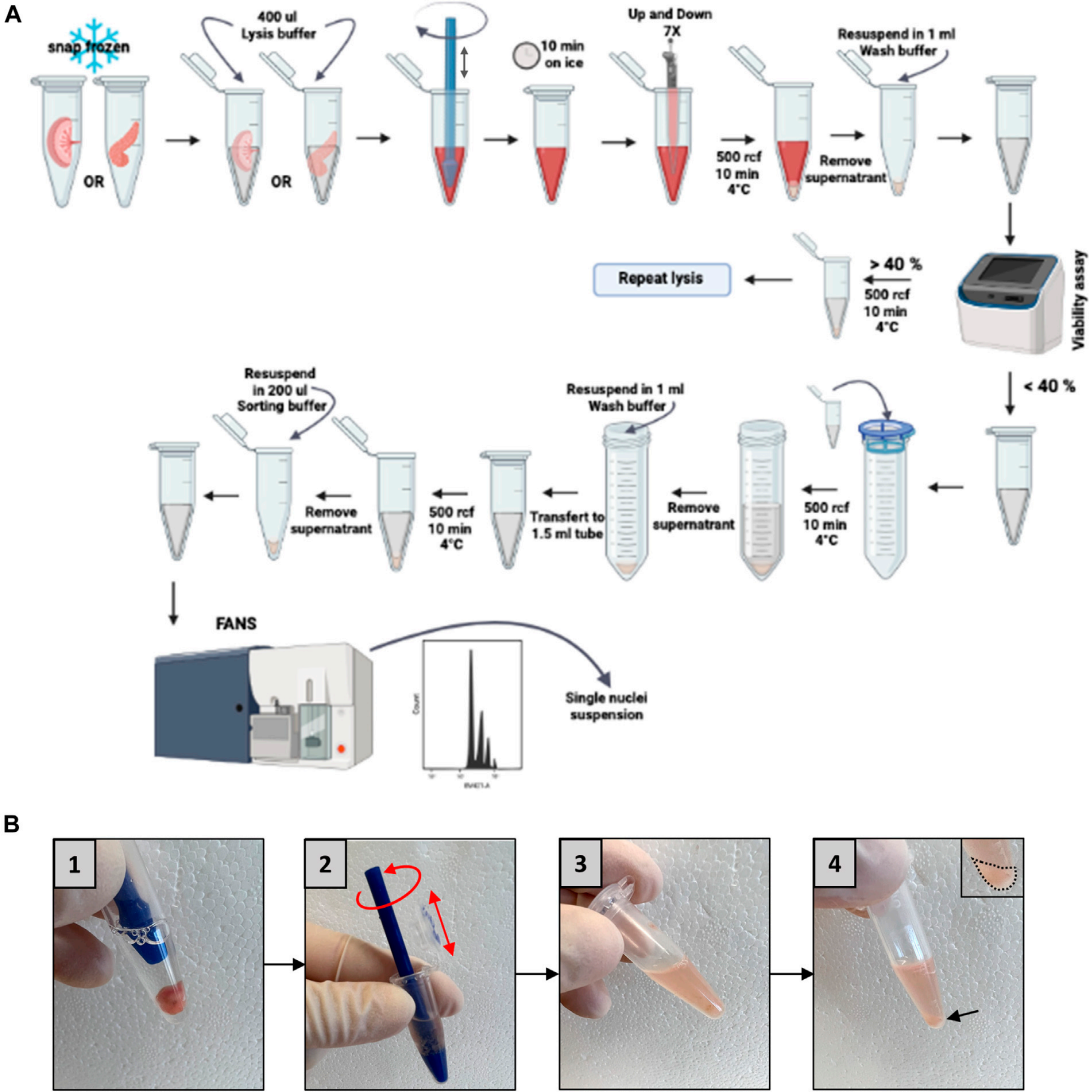
**Placenta or pancreas homogenization and nucleus isolation. (A)** Schematic representation of nucleus isolation protocol**. (B)** Representative pictures of the homogenization steps (5 strokes in a 1.5 mL Eppendorf, box numbers 1–3) and the nucleus pellet obtained (arrow, box number 4). FANS: fluorescent-activated nuclei sorting.

Figure 4 shows a detailed scheme of this step.

To minimize RNA degradation, all steps are carried out on ice. The homogenizer, Eppendorf tubes, and all buffers need to be pre-cooled. All resuspension steps are done by carefully pipetting the nucleus suspension. Do not vortex the sample to avoid shearing forces and damage to the nuclei.

1. Place the frozen 1.5 mL tube containing the tissue directly on ice and add very rapidly 600 μL lysis buffer into the tube.
• 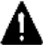
CRITICAL STEP: It is important to add the lysis buffer rapidly to avoid the degradation of the tissue.
2. Break the sample with the homogenizer to obtain a homogenous lysis of the tissues. For this purpose, take a sterile pestle with one hand and the Eppendorf in which the tissue sample is immersed in the lysis buffer with the other hand. Immerse the pestle in the buffer so that the sample is trapped between the end of the pestle and the bottom of the Eppendorf. Rotate the pestle continuously from right to left and up and down (10–15 movements, this number increasing with sample size) to dissociate the sample. Mechanical lysis is complete when the solution becomes cloudy and no more tissue fragments are visible.
• 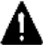
CRITICAL STEP: It is important to perform this step rapidly after adding the lysis buffer to avoid lysis only on the tissue periphery.
3. Incubate on ice 10 min and gently swirl to mix every 2 min.
4. Up and down gently 10x the lysate with the 1,000 μL regular bore pipette.
5. Centrifuge the sample at 500 rcf for 10 min at 4°C.
6. Remove the supernatant without disrupting the pellet.
7. Add 1 mL of wash buffer using a regular-bore pipette tip and gently do 10 up and down. If the resuspension of the pellet is difficult, use a 200 μL regular-bore pipette tip.
8. Assess the viability to evaluate the efficiency of lysis: take 5 μL of lysate and stain with trypan blue (1:1), put the stained solution in a counter chamber and use the counter cell to evaluate the viability.
• If the viability is more than 40%, centrifuge at 500 rcf for 10 min at 4°C and repeat the lysis step (steps 1–7).
9. Put a 40 μm filter on a 50 mL Falcon tube and pass the 1 mL lysate through the filter. Rinse the 1.5 mL Eppendorf with 1 mL wash buffer and pass through the filter.
10. Add 3 mL of wash buffer to the 50 mL Falcon tube.
11. Centrifuge at 500 rcf for 10 min at 4°C and remove the supernatant.
12. Resuspend the pellet by adding 1 mL of wash buffer using a 1,000 μL regular-bore pipette tip and gently do 5 up and down. Then, perform 10 up and down with a 200 μL regular-bore pipette.
13. Transfer the solution to a new 1.5 mL tube.
14. Centrifuge at 500 rcf for 10 min at 4°C and remove the supernatant.
15. Add 500 μL of sorting buffer (wash buffer + DAPI) using a regular-bore pipette tip and gently up and down 10x.

Visually inspect nuclei on a hemocytometer to assess morphology, damage, and aggregation, and verify the DAPI staining using a fluorescent microscope.

### 3.4 Fluorescent-activated nucleus sorting—± 30 min

**FIGURE 5:**
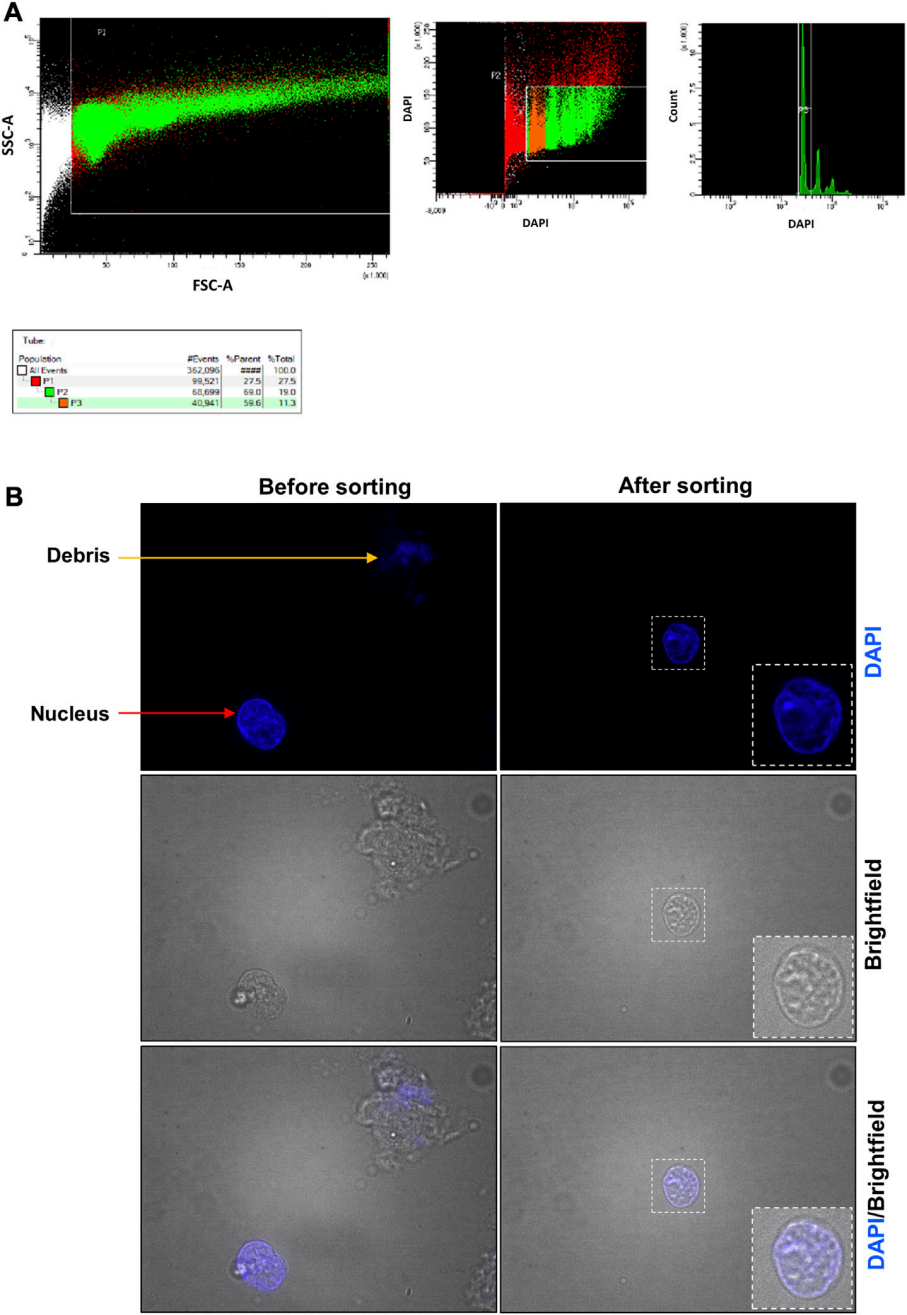
**Placenta fluorescent-activated nucleus sorting and quality control assessment**. **(A)** Gates used to identify the placental nuclei. In the left panel, the designed window excludes debris and selects events that were distributed according to their internal content and size on the SSC-A and FSC-A axes, respectively. In the middle panel, events were represented according to their level of DAPI staining. Nuclei are represented in orange (2n nuclei, P3 selection) and in green (2n, 4n, … nuclei, P2 selection). Red events are considered not to be nuclei. In the right panel, events were represented according to the number of count and their level of DAPI staining. Most nuclei were 2n. **(B)** Nucleus suspension obtained before and after the sorting. Before the sorting, the nucleus suspension contains cell debris (orange arrow), nuclei (red arrow) and aggregates (not shown). After the sorting, the nucleus suspension is cleaner and contains essentially nuclei.

**FIGURE 6:**
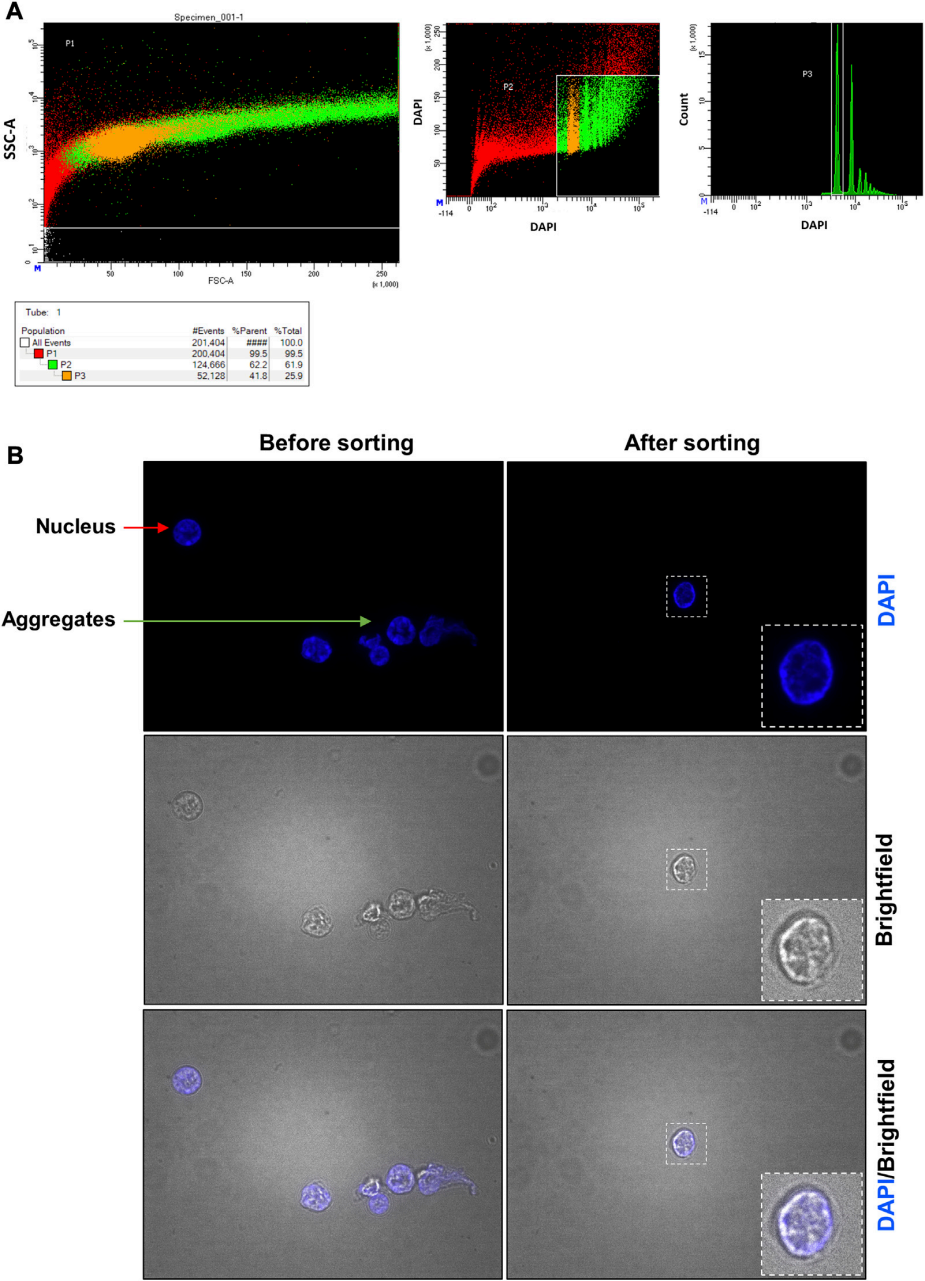
**Pancreas fluorescent-activated nucleus sorting and quality control assessment**. **(A)** Gates used to identify pancreatic nuclei. In the left panel, the designed window excludes debris and selects events that were distributed according to their internal content and size on the SSC-A and FSC-A axes, respectively. In the middle panel, events were represented according to their level of DAPI staining. Nuclei are represented in orange (2n nuclei, P3 selection) and in green (2n, 4n, … nuclei, P2 selection). Red events are considered not to be nuclei. In the right panel, events were represented according to the number of count and their level of DAPI staining. Two red peaks are obtained representing the two major different DNA ploidies of the nuclei (2n or 4n): most nuclei were 2n. **(B)** Nucleus suspension obtained before and after the sorting. Before the sorting, the nucleus suspension contains cell debris (not shown), nuclei (red arrow) and aggregates (green arrow). After the sorting, only nuclei are found.

NOTE Figures 5, 6 illustrate the fluorescent-activated nucleus sorting of the two different organs and the quality control of the nuclei post FANS. Before starting the sorting, calculate the required volume of nucleus suspension for the desired recovery of sequencing. The optimal concentration is between 700 and 1,200 nuclei/μL.

1. Perform the sorting procedure at 4°C.
2. Load the 1.5 mL tube containing the sample into the cell sorter.
3. For sorting, use the following parameter: nozzle 100 μm.
4. Sort the nuclei into a 1.5 mL Eppendorf containing wash buffer.
5. When the number of sorted nuclei reaches the previously defined number, you can stop the sorting.
6. To analyze the quality of the sorted nuclei, take a small volume of the nucleus suspension and verify under fluorescence microscope the shape of nuclei.
• Nuclei of good quality appear round and smooth with an intact membrane and are well-separated.
7. To avoid nucleus aggregation and RNA degradation, proceed immediately to library preparation.

### 3.5 Single nucleus RNA sequencing

The efficiency of the protocol was evaluated by performing snRNA-seq using the 10x Genomics technology. The library preparation was performed using the 10x Chromium Single Cell 3’ Gene Expression Kit v3.1 and following the chromium 10x V3 protocol.

SnRNAseq data was processed with CellRanger version 7.1.0. 84% of the nuclei had less than 0.25% of mitochondrial genes, suggesting high quality and successfully stripped nuclei. The number of UMIs and the number of detected genes respectively ranged from 501 to 185817 and from 263 to 13148 (with a median values of 7,341 and 2860), indicating that the sequencing quality was good and suggesting that transcript diversity was successfully captured.

## 4 Anticipated results and discussion

Fluorescent-activated nucleus sorting allows the obtention of a concentrated and clean single-nucleus suspension.

After lysis of the frozen organ (placenta or pancreas), the sample is a mixture of single nuclei, cell debris, and aggregates (Figure 5B; Figure 6B before sorting). To remove all contaminants and obtain a single nucleus suspension, we used flow cytometry.

For the placenta sample (Figure 5), a fluorescent-activated nuclei sorting (FANS) (Figure 5A) was performed to obtain a clear suspension composed by only single nuclei. Between 6 × 10^5^ and 1.3 × 10^6^ nuclei can be purified from 100 mg of placenta. After flow cytometry, we visualized the sorted nuclei under fluorescence microscope to ensure that it contains only single nuclei with good morphology. The quality control after FANS confirmed that it was the case (Figures 5B—after sorting).

For the pancreas sample (Figure 6), FANS (Figure 6A) was performed to obtain a pure suspension containing only single nuclei. Between 10^6^ and 1.7 × 10^6^ nuclei can be purified from 100 mg of pancreas. To assess the impact of the sorting technique on the quality of nuclei and its efficiency, we visualized the sorted nuclei under fluorescence microscope. This confirmed the validation of the preservation of a good nucleus morphology and the absence of aggregates using DAPI staining (Figures 6B—after sorting).

### 4.1 The use of frozen tissue preserves the quality of the sequencing

**FIGURE 7:**
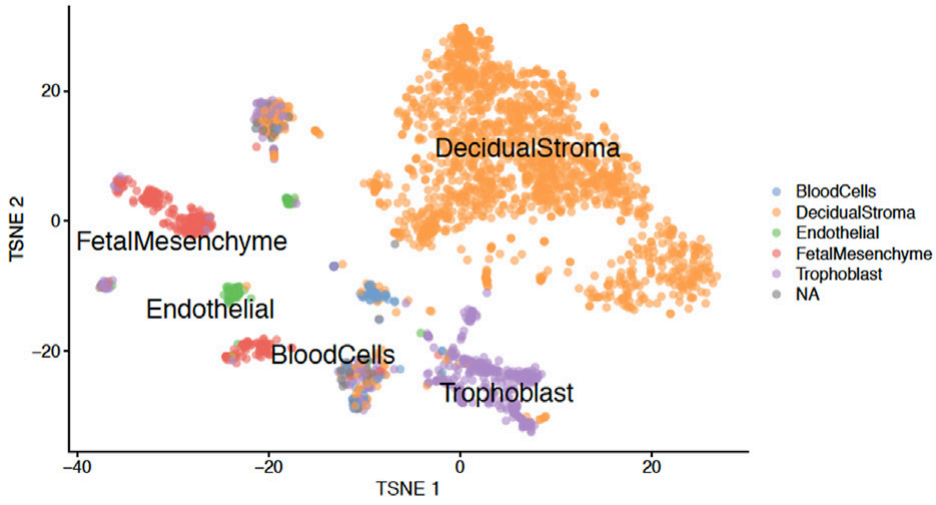
**T-SNE plot.** Graph showing the clustering of placenta nuclei between different cell types after analysis of sequencing data.

The efficiency of our protocol was evaluated by performing snRNA-seq on the placenta nuclei using the 10x Genomics technology. The sequencing data allowed us to generate a T-SNE plot and identify nucleus clusters corresponding to the cell types found in the placenta (Figure 7).

To be sure that our protocol of nucleus isolation from frozen tissues works correctly and does not introduce artefact in the generated sequencing data, we compared our results with the data obtained from fresh placenta samples (Marsh and Blelloch, 2020). The clustering of our nuclei based on transcriptomic data allows us to obtain six clusters (Figure 7) corresponding to the decidual stroma, fetal mesenchyme, trophoblast cells, endothelial cells, blood cells and NA (non-attributed). This is very similar to the Marsh and Blelloch’s data which identified the same clusters, except for the NA cluster. Thus this comparison confirms the efficiency of our protocol.

### 4.2 Problems that can be solved

We will discuss here the most frequently encountered problems, and the way in which they can be solved, knowing that the absence of use of commercial kits in our protocol will allow easier resolution of these problems. Thus, it happens that too few nuclei are obtained. This problem generally finds its origin either in the step of mechanical dissociation of the frozen tissue (Figure 4B, picture 2), or in the step of lysis of the cells. A mechanical dissociation problem is manifested by the persistence of large tissue fragments after homogenization. It is explained by the fact that the lysis solution did not penetrate into the whole sample, and that the lysis was therefore not homogeneous. This can be seen with more fibrous tissues which might be present in transgenic mice, for example. In this case, it is necessary to increase the number of movements of the pestle, the precise parameters having to be determined on a case-by-case basis by the user, depending on the extent of the fibrosis.

Furthermore, if a low concentration of nuclei is obtained after sorting, this will be a problem for the generation of library by 10X Genomics technology. Indeed, a low concentration of nuclei will not allow the loading of the desired number of nuclei into the 10X Genomics chip. To solve this, it is important to sort the maximum number of nuclei, and after centrifugation of the sorted nuclei, to resuspend the nucleus pellet in the smallest possible volume. The resuspension volume will depend on the number of sorted nuclei obtained and the concentration of nuclei desired for the chip loading.

## 5 Conclusion, strengths, and limitations

In summary, the present protocol is an efficient way to obtain a single nucleus suspension from frozen tissues. A significant advantage of this protocol is that, beyond placenta and pancreas, it could be applied to other frozen organs. Moreover, the protocol allows to obtain many nuclei with a good morphology in a short time. A limitation is that snRNA-seq will not provide data on the possible presence of splicing isoforms, as nuclear RNAs still exhibit their introns.

Compared to other protocols, we believe that our protocol offers a unique combination of advantages, namely, the use of frozen tissues, laboratory-made buffers, and a protocol for purification of nuclei by FANS. These advantages are reflected in practice by obtaining better QC parameters after sequencing. Thus, a comparison with a protocol from a biotechnology company (https://pages.10xgenomics.com/rs/446-PBO-704/images/10x_LIT000163_Product_Sheet_Nuclei_Isolation_Kit_Letter_digital.pdf), which notably uses isolation column to obtain the nuclei, shows that we obtain a mean number of detected genes approximately 3 times higher. Compared to a protocol that uses a filter to remove remaining debris (Kimbley et al., 2022), we also obtain significantly less mitochondrial gene contamination. The automatic counting of nuclei during FANS also allows us to save precious time to preserve the integrity of the RNAs, in comparison to a protocol that uses a disposable hemacytometer for counting nuclei (Rousselle et al., 2022). Finally, the FANS can also be credited with saving time, compared to a protocol that uses a sucrose gradient for the nucleus clean-up (Stalder et al., 2023).

## Data Availability

The data presented in the study are deposited in NCBI’s Gene Expression Omnibus and are accessible through GEO Series accession number GSE243258.
