# Supplementary material for: Optimized nucleus isolation protocol from frozen mouse tissues for single nucleus RNA sequencing application
Source: Front Cell Dev Biol. 2023 Sep 28;11:1243863. doi: 10.3389/fcell.2023.1243863 (PMC10575574; doi:10.3389/fcell.2023.1243863)
Supplement: Supplementary file 1 [file Table1.pdf]

| | Stock concentration | Final concentration | Volume |
|---|---|---|---|
| **Lysis Buffer** | | | **10 ml** |
| Tris-HCL (pH 7.2-7.4) | 1M | 10 mM | 100 µl |
| NaCl | 5M | 10 mM | 20 µl |
| $MgCl_2$ | 1M | 3 mM | 30 µl |
| Nonidet P40 Subsitute | 10 % | 0.1 % | 100 µl |
| Nuclease-free water | - | - | 9.75 ml |
| | | | |
| **Wash Buffer** | | | **50 ml** |
| BSA powder | | 1 % | 0.5 g |
| RNase Inhibitor | 40 U/µl | 0.1 U/µl (to 0.5 U/µL) | 125 µl |
| 1X PBS | - | - | 39.875 ml |
| | | | |
| **Sorting Buffer** | | | **2 ml** |
| DAPI | 5 mg/ml | 10 µg/ml | 4 µl |
| Wash buffer | - | - | 1.96 ml |

**Suppl. Table 1.** Solutions to be prepared.
